# Hemorrhagic Vocal Fold Inflammation Following Endotracheal Intubation in a Patient Undergoing Endovascular Neurosurgery Under Dual Antiplatelet Therapy: A Case Report

**DOI:** 10.1155/cria/9524667

**Published:** 2026-10-28

**Authors:** Masayoshi Koike, Takako Terui, Tomohiro Nakanishi, Risa Tuboi, Hiroyuki Enomoto, Toshihiro Ishibashi

**Affiliations:** ^1^ Department of Anesthesiology The Jikei University School of Medicine Tokyo Japan jikei.ac.jp; ^2^ Division of Endovascular Neurosurgery Department of Neurosurgery The Jikei University School of Medicine Tokyo Japan jikei.ac.jp

**Keywords:** dual antiplatelet therapy, endotracheal intubation, endovascular neurosurgery, hemorrhagic vocal fold inflammation, vocal fold hemorrhage

## Abstract

**Background:**

Hemorrhagic vocal fold inflammation is an uncommon complication of endotracheal intubation. Its clinical course in patients receiving perioperative antithrombotic therapy remains unclear.

**Case Presentation:**

A woman in her 60 s receiving dual antiplatelet therapy (DAPT) underwent endovascular neurosurgery with flow‐diverter stent deployment and coil embolization. Endotracheal intubation was uneventful, and cuff pressure was appropriately maintained. Hoarseness developed on postoperative day 1, and laryngoscopy on day 2 revealed hemorrhagic vocal fold inflammation. Intraoperative fluoroscopic images suggested proximal migration of the endotracheal tube, with possible changes in tube and cuff position that may have contributed to minor vocal fold mucosal injury. The symptoms improved with conservative management, and complete recovery was confirmed at 1 month.

**Discussion:**

Hemorrhagic vocal fold inflammation occurred following apparently uneventful endotracheal intubation in a patient receiving perioperative antithrombotic therapy. Although the underlying mechanism remains uncertain, changes in endotracheal tube and cuff position during the procedure represent one possible contributing factor.

## 1. Introduction

Endotracheal intubation is routinely performed during general anesthesia, and most airway‐related complications are minor and transient. However, hemorrhagic vocal fold inflammation, although uncommon, may lead to clinically significant symptoms such as hoarseness and dysphonia.

The reported incidence of vocal fold hemorrhage following endotracheal intubation is approximately 4% [1], but the influence of perioperative antithrombotic therapy on its severity remains unclear.

We report a case of hemorrhagic vocal fold inflammation following endotracheal intubation in a patient undergoing endovascular neurosurgery under dual antiplatelet therapy (DAPT) with intraoperative anticoagulation.

## 2. Case Presentation

A woman in her 60 s (height 163 cm and weight 48 kg) underwent endovascular coiling with adjunctive flow‐diverter stent placement for an unruptured right internal carotid artery aneurysm (neck 4.11 mm, height 4.34 mm, and width 5.05 mm).

### 2.1. Medical History

Her medical history included dyslipidemia treated with rosuvastatin and Hashimoto’s disease treated with levothyroxine. For the prevention of thromboembolic complications after endovascular surgery, DAPT (aspirin and clopidogrel) had been initiated 2 weeks before surgery.

### 2.2. Anesthetic Management

General anesthesia was induced with fentanyl, remifentanil, propofol, and rocuronium. Endotracheal intubation was performed using a McGrath MAC video laryngoscope with a size 3 blade. A 7.0‐mm internal diameter endotracheal tube was placed without difficulty on the first attempt. The endotracheal tube was secured at the left corner of the mouth as directed by the surgeon and then secured at a length of 19 cm. Cuff pressure was continuously monitored and maintained between 20 and 30 cmH_2_O. The total duration of surgery was 2 h and 52 min, with an anesthesia time of 3 h and 46 min and an endotracheal intubation time of 3 h and 33 min.

Intraoperatively, intermittent heparin administration was used to maintain an activated clotting time (ACT) above 250 s. The procedure was completed uneventfully, and the patient was extubated smoothly without severe coughing and transferred to the ICU.

Postoperatively, continuous intravenous infusion of heparin was initiated to prevent thrombotic complications. Perioperative anticoagulation and antiplatelet therapy, along with serial monitoring values including ACT, APTT, PT‐INR, and ADP‐induced platelet aggregation, are shown in Figure 1. The data indicate that the patient was in a markedly anticoagulated and antiplatelet state due to 2 weeks of preoperative DAPT and intraoperative heparin administration.

**FIGURE 1:**
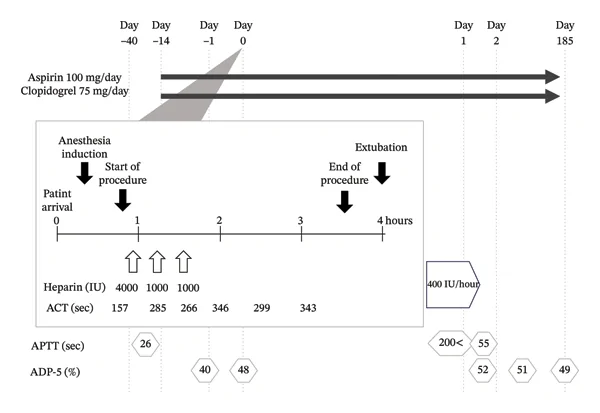
Perioperative anticoagulation and antiplatelet management. Figure 1 provides an overview of the perioperative anticoagulation and antiplatelet regimen, together with serial monitoring parameters including ACT, APTT, PT‐INR, platelet count, and the ADP‐induced platelet aggregation test. ADP‐5: ADP (5 μmol/L)‐induced platelet aggregation.

### 2.3. Postoperative Course

Continuous intravenous infusion of heparin was discontinued on the morning of the postoperative day (POD) 1. There was no complaint of hoarseness before discharge from the ICU on POD1. Hoarseness was subsequently noted later that evening. On POD2, the patient reported marked hoarseness and throat pain. Otolaryngological consultation was requested, and fiberoptic laryngoscopy revealed hemorrhagic vocal fold inflammation (Figure 2). As no active bleeding was observed, conservative management was recommended. Symptoms gradually improved, and both clinical and laryngoscopic findings had significantly resolved by POD6 (Figure 3). Complete recovery was confirmed at 1‐month follow‐up (Figure 4). As shown in Figure 4, the hemorrhage had nearly resolved at the 1‐month follow‐up, and although a small vocal fold polyp was still observed, the patient’s hoarseness and other related symptoms had completely resolved.

**FIGURE 2:**
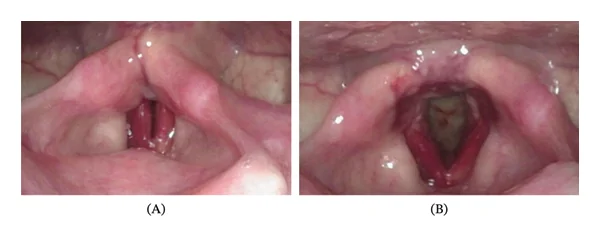
Fiberoptic findings on postoperative day 2 (A, B) Fiberoptic images obtained during an ENT consultation on postoperative day 2 show marked erythema and swelling of the vocal folds. These findings are consistent with hemorrhagic vocal fold inflammation. No identifiable active bleeding point was observed, and conservative observation was recommended.

**FIGURE 3:**
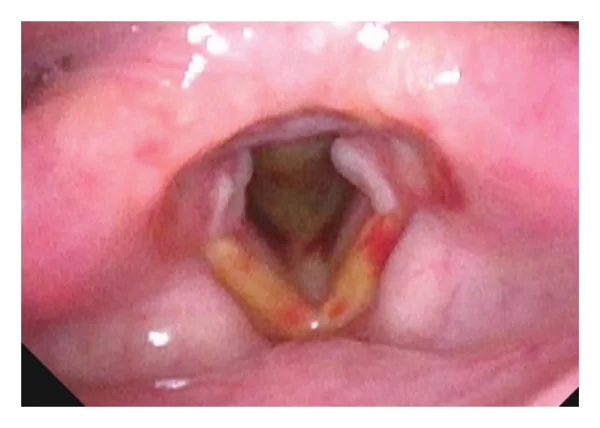
Fiberoptic findings on postoperative day 6. Hoarseness and throat pain had improved by postoperative day 6. Follow‐up fiberoptic evaluation performed by the ENT specialist showed gradual improvement in vocal fold erythema and swelling, consistent with ongoing clinical recovery.

**FIGURE 4:**
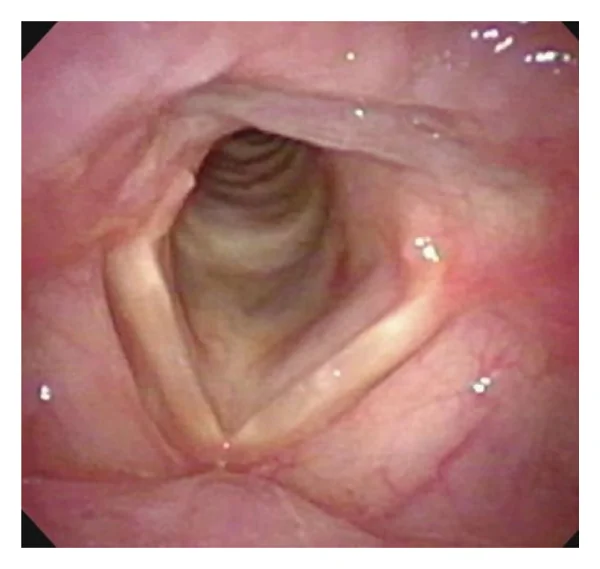
Fiberoptic findings at the 1‐month follow‐up. At the 1‐month follow‐up visit, the patient’s hoarseness and throat pain had fully improved. Fiberoptic laryngoscopy performed by the ENT specialist demonstrated complete resolution of the previously observed vocal fold erythema and swelling, confirming full recovery from hemorrhagic vocal fold inflammation.

At the 3‐month follow‐up, the previously noted small vocal fold polyp had completely resolved, and no residual laryngeal abnormalities were observed.

DAPT was continued because no progression of hemorrhage was observed and prevention of thrombus formation was critical.

Following this episode, intraoperative fluoroscopic images were reviewed. Initially, the depth of the endotracheal tube was adjusted so that the depth‐marking lines aligned with the vocal cords; however, the tube may have subsequently become shallower during the procedure. Indeed, intraoperative fluoroscopic images (Figure 5) suggested proximal migration of the endotracheal tube. Although the position of the cuff relative to the vocal folds could not be determined, the observed tube migration raises the possibility that changes in tube and cuff position may have contributed to minor mucosal injury of the vocal folds.

**FIGURE 5:**
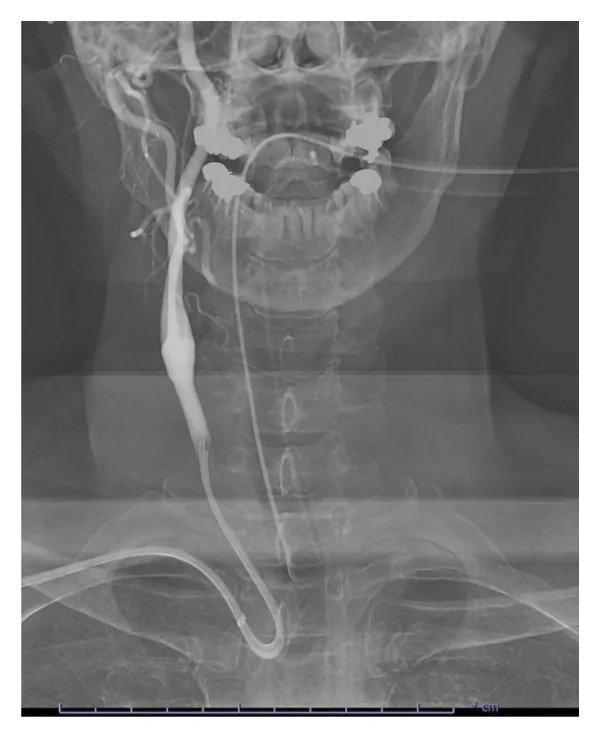
Intraoperative fluoroscopic images. Intraoperative fluoroscopic images obtained during the endovascular neurosurgery show the position of the endotracheal tube. The tube tip appears to have migrated proximally, raising the possibility that the cuff may have approached the level of the vocal folds.

## 3. Discussion

Hemorrhagic vocal fold inflammation is a rare condition that can cause hoarseness and, in severe cases, airway obstruction. In the present case, factors related to endotracheal intubation and airway management, together with the effects of perioperative antiplatelet and anticoagulant therapy, may have contributed to its development. Although endotracheal intubation was performed smoothly and the endotracheal tube was initially positioned appropriately, its depth may have changed during the procedure. In neuroendovascular procedures requiring perioperative antiplatelet and anticoagulant therapy, intraoperative fluoroscopic images may provide an opportunity to reassess endotracheal tube position.

In the present case, the endotracheal tube was inserted from the right side of the oral cavity, and the depth was adjusted so that the two depth‐marking lines aligned with the vocal cords [2]. The endotracheal tube was then repositioned to the left oral commissure and secured as directed by the surgeon. As shown in Figure 5, the endotracheal tube appeared to be positioned on the right side at the pharyngeal level. This finding raises the possibility that the change in the course of the endotracheal tube, either after intubation or following the start of surgery, resulted in relative proximal displacement of the tube tip. Changes in the endotracheal tube tip position associated with repositioning of the endotracheal tube have been reported [3], and a similar phenomenon may have occurred in this case. Although several studies have described changes in tube tip position caused by head and neck movement [3, 4], those reports focusing on changes in the tube tip position due to repositioning of the tube while the head remains in a neutral position are limited. In this case, the anesthesia equipment was positioned at the foot end of the operating table. Therefore, the influence of circuit positioning on tube tip displacement warrants further investigation.

Previous reports indicate that vocal fold hemorrhage occurs in approximately 4% of patients after endotracheal intubation [1]. Other potential contributing factors include phonotrauma, severe coughing, and pharyngitis; however, none of these were present in this case [5, 6]. No studies have specifically evaluated the incidence or severity of vocal fold hemorrhage in patients receiving perioperative antiplatelet or anticoagulant therapy such as DAPT or heparin. Although granuloma formation associated with shallow endotracheal tube positioning has been reported [7], hemorrhagic vocal fold inflammation following endotracheal intubation has rarely been described. Vocal fold or laryngeal hematoma has also been reported in association with anticoagulant therapy [8, 9], with one reported case resulting in airway obstruction requiring endotracheal intubation [8]. Furthermore, the potential influence of perioperative antiplatelet and anticoagulant therapy on vocal fold hemorrhage associated with minor mucosal injury after endotracheal intubation has not been sufficiently investigated.

Although previous reports have described the development of hoarseness and laryngeal granulomas after prolonged endotracheal intubation [10], the intubation time in the present case was 3 h and 33 min. A recent systematic review and meta‐analysis reported that vocal fold hematoma was uncommon and found no significant difference in its occurrence between procedures lasting < 120 min and ≥ 120 min [11]. Thus, the occurrence of hemorrhagic vocal fold injury may not necessarily be related to prolonged intubation alone. In the present case, perioperative antiplatelet and anticoagulant therapy may have increased susceptibility to bleeding from minor mucosal injury. Hoarseness was not documented on the day of surgery, and the medical record did not clearly establish whether it was present or absent. Vocal fold hemorrhage may be asymptomatic or may present with gradually progressive voice symptoms [5]. Therefore, the symptoms may initially have been inapparent, or the hemorrhagic changes may have evolved over time. Accordingly, the timing of symptom recognition alone does not establish or exclude endotracheal tube‐ or cuff‐related trauma as a contributing mechanism.

This case has several limitations. Direct confirmation of the relationship between the cuff and the vocal folds was not available because neither bronchoscopic nor laryngoscopic evaluation was performed intraoperatively. At the time of surgery, however, there was no clinical indication for additional airway evaluation because endotracheal intubation was uneventful, ventilation remained stable, and cuff pressure was appropriately maintained throughout the procedure. Although intraoperative fluoroscopic images suggested proximal migration of the endotracheal tube, the position of the cuff relative to the vocal folds could not be determined. Therefore, cuff contact with the vocal folds and its potential contribution to mucosal injury remain hypothetical in this case.

The patient had been on DAPT for 2 weeks preoperatively, received intraoperative heparin, and continued DAPT together with postoperative anticoagulation (heparin). As shown in Figure 1, marked prolongation of ACT and APTT was observed during and after the procedure due to intraoperative and postoperative heparin use, indicating that the patient remained in a highly hemorrhagic state until continuous heparin infusion was discontinued. In addition, the ADP‐5 value was mildly reduced preoperatively, reflecting the antiplatelet effect of ongoing DAPT. Taken together with intravenous anticoagulation, these findings suggest that the patient was at a markedly increased risk of bleeding.

Recent institutional experience at our center indicates that maintaining ADP‐5 values within a target range of 40%–60% is optimal for balancing the prevention of thromboembolic complications after stent‐assisted coil embolization while minimizing bleeding risk [12]. In this case, the patient’s ADP‐5 values were appropriately controlled within this therapeutic window, suggesting that antiplatelet therapy alone did not induce excessive platelet inhibition. The transient vocal fold hemorrhage may therefore have been attributable to the additive anticoagulant effects of intraoperative and postoperative heparin. Mechanistically, DAPT inhibits platelet activation through suppression of thromboxane A2 production and ADP‐mediated platelet activation, thereby impairing platelet plug formation and stability, whereas heparin suppresses the coagulation cascade [13]. Although the relative contribution of each therapy cannot be determined, these distinct effects on hemostasis may both have influenced the vocal fold hemorrhage. Conversely, the relatively rapid clinical recovery may have been facilitated by the fact that platelet aggregation was not profoundly suppressed, allowing mucosal hemostasis and healing to proceed once the heparin effect diminished. This intensive antithrombotic milieu may have exacerbated the severity of the vocal fold hemorrhage. Furthermore, contact of the endotracheal tube or cuff with the vocal folds may have caused mechanical irritation, with subsequent edema and ischemic changes potentially contributing to the evolution of the vocal fold injury.

In this case, clinically apparent hemorrhagic vocal fold inflammation occurred despite apparently uneventful endotracheal intubation and appropriate cuff pressure management. Although the causal relationship between endotracheal tube position and this laryngeal injury remains uncertain, this case suggests that changes in endotracheal tube and cuff position may occur during the procedure without obvious clinical signs. In endovascular neurosurgery, routinely obtained intraoperative fluoroscopic images may provide an opportunity to identify otherwise unrecognized changes in endotracheal tube position. Careful assessment of these images, together with attention to possible changes in endotracheal tube position throughout the procedure, may help minimize tube‐related laryngeal injury.

In conclusion, this case describes hemorrhagic vocal fold inflammation following apparently uneventful endotracheal intubation in a patient receiving perioperative antithrombotic therapy. Although the underlying mechanism remains uncertain, minor mucosal injury related to intubation or changes in endotracheal tube and cuff position may have contributed to the development of hemorrhagic vocal fold inflammation.

**Nomenclature**
POD: Postoperative day
DAPT: Dual antiplatelet therapy
ACT: Activated clotting time
ICU: Intensive care unit
ENT: Ear, nose, and throat

## Author Contributions

Masayoshi Koike drafted the manuscript. Tomohiro Nakanishi, Risa Tuboi, Hiroyuki Enomoto, and Toshihiro Ishibashi collected clinical data. Masayoshi Koike and Takako Terui revised and edited the manuscript.

## Funding

This research received no specific grant from any funding agency in the public, commercial, or not‐for‐profit sectors.

## Disclosure

All authors read and approved the final version of the manuscript.

## Ethics Statement

Ethical approval was not required for this single‐patient case report, in accordance with institutional policy.

## Consent

Written informed consent for publication of this case report and accompanying images was obtained from the patient.

## Conflicts of Interest

The authors declare no conflicts of interest.

## Data Availability

The data supporting the findings of this case report are available within the article. No additional datasets were generated or analyzed.
